# Effect of the addition of microcapsules with avocado peel extract and nisin on the quality of ground beef

**DOI:** 10.1002/fsn3.1359

**Published:** 2020-02-22

**Authors:** Mariel Calderón‐Oliver, Héctor B. Escalona‐Buendía, Edith Ponce‐Alquicira

**Affiliations:** ^1^ Tecnologico de Monterrey Escuela de Ingeniería y Ciencias Toluca de Lerdo Mexico; ^2^ Departamento de Biotecnología Universidad Autónoma Metropolitana Iztapalapa Mexico

**Keywords:** avocado peel extract, microcapsules, minced meat, nisin, oxidation prevention

## Abstract

This study evaluated the incorporation of microcapsules containing nisin and avocado peel extract on the shelf life of ground beef. Ten treatments were studied and divided into two groups: one packaged under vacuum and the other in permeable packaging. Each group contained: (a) control, (b) extract, (c) nisin, (d) empty microcapsules (only wall microcapsule system), and (e) microcapsules with extract and nisin. The samples containing the microcapsules presented lower bacterial growth and less oxidation. On day 10, the vacuum‐packaged samples with microencapsulated preservative presented a reduction in the oxidation of proteins of approximately 45%, of 30% in the growth of mesophiles, and of 38% in the growth of coliforms, as well as a reduction in the changes in the pH and *ɑ*_W_ that occur during storage, compared with the permeable control. The combination of microcapsules with vacuum packaging reduced the physicochemical and microbiological changes that occur in the controls.

## Graphical abstract

Microcapsules with natural preservatives prevent deterioration changes in meat. Encapsulation improved the preservatives properties of nisin and avocado peel. Microencapsulation improves microbial and oxidation stability of minced meat.

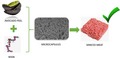

## 1 INTRODUCTION

Meat and meat products are susceptible to oxidation and microbiological deterioration. Lipid content, the presence of iron, type of meat, and other factors are involved in oxidation reactions, while the protein content and the addition of carbohydrates encourage the growth of pathogens and altering bacteria (Falowo, Fayemi, & Muchenje, 2014; Guyon, Meynier, & de Lamballerie, 2016; Simpson & Sofos, 2009). In particular, the growth of pathogens, such as *Listeria monocytogenes*, *Salmonella*, *Escherichia coli*, and other undesirable microorganisms, is considered a health risk for consumers (Nørrung, Andersen, & Buncic, 2009).

Additionally, the oxidation of lipids and proteins in meat decreases the nutritive value of the meat because some important amino acids, such as proline, lysine, and arginine, are oxidized into carbonyl residues (Zhang, Xiao, & Ahn, 2013); also, the oxidation of the protein myoglobin produces changes in color (Faustman, Sun, Mancini, & Suman, 2010). In the case of lipids, their oxidation produces molecules such as aldehydes that give meat rancid odors and off flavors (Kanner, 1994).

For that reason, multiple strategies have been implemented to extend the shelf life of meats and other foods and to reduce undesirable reactions. These strategies include the inclusion of antioxidants and antimicrobials, for which consumers encourage the use of those from natural origins (Falowo et al., 2014; Salehi, Tumer, et al., 2019; Sharifi‐Rad et al., 2018), also if they have health benefits (Salehi, Sharifi‐Rad, et al., 2019; Salehi, Vlaisavljevic, et al., 2019). A good source of antioxidants and antimicrobials are agro‐industrial residues, such as the seed and peel of fruits (Ayala‐Zavala et al., 2011). Avocado peel extract has been demonstrated to have an antioxidant capacity because of its polyphenol content, and there have been reports about its antimicrobial activity or synergic effect with other natural antimicrobials, such as nisin (Calderón‐Oliver et al., 2016; Fu et al., 2011; Rodríguez‐Carpena, Morcuende, Andrade, Kylli, & Estevez, 2011).

However, the addition of some preservatives, such as polyphenols in food, produce sensorial changes, could interact with other compounds, reducing their effectiveness as antioxidants, or could be degraded by proteases, which is the case for nisin in meat (Fang & Bhandari, 2010; Liu & Hansen, 1990). The encapsulation process of those preservatives decreases their interaction with the matrix of the food, masks undesirable odors or flavors, and regulates their liberation into the medium (Shahidi & Han, 1993). Microencapsulation by complex coacervation is a method to encapsulate various compounds or probiotics with good encapsulation efficiency, ensuring the release of the bioactive compound (Comunian et al., 2013; Eratte et al., 2015). The complex coacervation involves the interaction of two biopolymers with opposite charge to form the capsule matrix (Gouin, 2004). In previous work, the elaboration and characterization of microcapsules made with this technique were reported, and the collagen–pectin system in a proportion of (1:1) with the spray dryer process had a higher encapsulation efficacy of nisin and avocado peel extract (84.66 ± 1.2 and 82.96 ± 1.25%, respectively) (Calderón‐Oliver, Pedroza‐Islas, Escalona‐Buendía, Pedraza‐Chaverri, & Ponce‐Alquicira, 2017). Therefore, this study aims to evaluate the effect of microcapsules containing a mixture of avocado peel extract and nisin (an antioxidant plus an antimicrobial) on the quality of minced meat. This evaluation considers the study of three factors (time, type of package, and addition microcapsules) on the physicochemical and microbiological responses in minced meat.

It is important to mention that the composition of the avocado peel extract, the optimization of the nisin‐extract mixture, and the microencapsulation system is already previously reported by our group (Calderón‐Oliver et al., 2016, 2017).

## 2 MATERIALS AND METHODS

### 2.1 Chemicals

Nisin Z (2.5% w/w balanced with sodium chloride and denatured milk solids, 10^6^ IU/g) was provided by Handary S.A. Avocado peel extract with antioxidant activity (ORAC value of 285.18 µg Trolox equivalents/mg) was extracted according to a previous study (Calderón‐Oliver et al., 2016). Hydrolyzed collagen (Peptan 5000) was purchased from Rousselot. Partially amidated low‐methoxyl pectin (standardized with sucrose, Genu LM‐104 AS) was purchased from CP Kelco. The minced beef meat was purchased in a local market with TIF certification (Mexican safety and health inspection regulation certification). The meat samples were transported and stored in refrigeration (4°C) until use. The composition of meat samples was measured according to the Association of Official Analytical Chemists methods (air‐oven for moisture, Kjeldahl method for crude protein, Soxhlet method with ether petroleum extraction for lipid content and ashes with weight difference after incineration at 525°C for 4 hr (AOAC, 1998)). The chemical composition of the meat was as follows: protein 18.89 ± 0.75%, lipids 6.58 ± 0.65%, moisture 73.47 ± 0.09%, and ashes 1.27 ± 0.01%.

### 2.2 Microcapsules preparation

Microcapsules were prepared by complex coacervation according to the method previously reported by our group (Calderón‐Oliver et al., 2017). Briefly, a W/O emulsion (1:2 ratio) that contained 0.15 g/ml of avocado peel extract and 0.1 g/ml of nisin was mixed with a solution of collagen 1% (w/v) and pectin 1% (w/v); then, the pH was adjusted to 3. After 24 hr of storage, the solution was spray‐dried (140°C inlet air and 70°C outlet air). The encapsulation efficiencies of avocado peel and nisin in these microcapsules were 84.66 ± 1.20 and 82.96 ± 1.25%, respectively. The empty microcapsules were prepared using the method described above, without the addition of avocado peel extract and nisin.

### 2.3 Incorporation of microcapsules in meat

Ten treatments were studied to compare the effects of microcapsules in three independent batches of meat. Each treatment is described in Table 1, which includes a control (C), empty microcapsules (EM), avocado extract (AE), and nisin (N), and others with microcapsules that contain the extract and nisin (MAN). Five treatments were packaged under an oxygen permeable system (code as P), and the other five were packaged under vacuum conditions (code as V). The corresponding number of microcapsules, AE or N (Table 1), were added to 200 g of meat, mixed for 3 min using a kitchen aid mixer, and then packaged and stored at 4°C for up to 10 days. Independent samples were taken for analysis on the 1st, 3th, 6th, 8th, and 10th days. The microbiological analyses include the count of mesophiles, coliforms, and lactic acid bacteria. Physicochemical analyses include evaluation of pH, *ɑ*_W_, and oxidation of lipids and proteins. Each determination was evaluated in triplicate. Only one concentration of microcapsules was used because it incorporated the maximum amount of nisin allowed by the Codex Alimentarius for meat products (Codex Alimentarius, 2019).

**Table 1:** Treatment groups

Treatment	Package system	Code	Microcapsules (g)	Avocado peel extract (g)	Nisin (g)
Control	Permeable	CP	0	0	0
Avocado peel extract	Permeable	AEP	0	0.28	0
Nisin	Permeable	NP	0	0	0.2
Empty microcapsule	Permeable	EMP	6	0	0
Microcapsule with avocado peel extract and nisin	Permeable	MANP	6	0.28	0.2
Control	Vacuum	CV	0	0	0
Avocado peel extract	Vacuum	AEV	0	0.28	0
Nisin	Vacuum	NV	0	0	0.2
Empty microcapsule	Vacuum	EMV	6	0	0
Microcapsule with avocado peel extract and nisin	Vacuum	MANV	6	0.28	0.2

### 2.4 Microbiological analyses

Twenty‐five grams of meat from each treatment were added to 225 ml of sterile saline solution (at 0.85%) and homogenized on a Stomacher 400 circulator (Seward) at 230 rpm for 30 s. If necessary, seriated decimal dilutions were made from this solution. The count of total aerobic bacteria was determined using MC Media Pads (Merck) (AOAC, 1994), while the enterobacteria population was determined on selective pads from 3 M Petrifilm (3 M) (AOAC, n.d.) according to the manufacturer instructions. Lactic acid bacteria (LAB) were determined by cultivation on *Lactobacilli* MRS agar under anaerobic conditions using a GasPak jar (BD BBL, Becton, Dickinson, and Company, Franklin Lake, NJ). The results are expressed as Log CFU/g).

### 2.5 Physicochemical analysis

#### 2.5.1 pH

Ten grams of meat were added to 100 ml of distilled water and homogenized in an Ultra‐Turrax (IKA) at 7,000 rpm for 1 min. Then, the homogenate was filtered (Whatman #4), and the resultant filtrate was used for pH measurement using a potentiometer (Orion, Versa Star, Thermo Fisher Scientific) (Schilling et al., 2018).

#### 2.5.2 Water activity (*ɑ*_W_)

Water activity determination was made in a water activity meter (Aqualab 4TE, Decagon Devices) by placing approximately 2 g of meat in the sample cup and following the manufacturer's instructions (Capita, Álvarez‐González, & Alonso‐Calleja, 2018).

#### 2.5.3 Oxidation stability

##### Lipid oxidation (TBARS method)

Lipid oxidation was determined using the method described by Salih, Smith, Price, and Dawson, (1987) with some modifications. Five grams of meat were homogenized at 11,000 rpm for 2 min in 15 ml of 4% perchloric acid. Then, the homogenate was filtered and centrifuged at 1,157 *g* for 10 min at 4°C. Two milliliters of the supernatant was added to 2 ml of 80 mM 2‐thiobarbituric acid. The reaction was developed via incubation for 30 min at 100°C. The absorbance of the samples and the standard curve (1,1,3,3‐tetramethoxypropane in perchloric acid in concentrations of 1.5–24 μM) were measured at 530 nm. The results are expressed as mg of malondialdehyde (MDA)/kg.

##### Protein oxidation

Protein oxidation was determined by derivatization of total carbonyls with 2,4‐dinitrophenylhydrazine, according to the method described by Oliver, Ahn, Moerman, Goldstein, and Stadtman, (1987) with modifications. One gram of meat was homogenized with 10 ml of 100 mM buffer phosphates pH 7.4 and then centrifuged at 1,157 *g* for 5 min. The supernatant was divided into two aliquots of 0.2 ml, and 1 ml of 10% trichloroacetic acid was added to each aliquot to precipitate proteins. After centrifugation of 3,214 *g* for 5 min, one of the pellets was treated with 1 ml of 2 M HCl (for protein determination), and the second was treated with 1 ml of 2,4‐dinitrophenylhydrazine (0.2% in 2 M HCl) for carbonyls determination. Both aliquots were incubated for 1 hr at room temperature, in the dark. Then, 1 ml of 10% trichloroacetic acid was added and incubated for 10 min at 4°C and centrifuged at 10,000 *g* for 10 min at 4°C. The incubation with trichloroacetic was repeated twice. Each pellet was treated with 1 ml of ethanol‐ethyl acetate (1:1) and centrifuged at 10,000 *g* for 10 min at 4°C. This operation was repeated twice. The final pellets were dissolved with 1 ml of 6 M guanidine HCl (pH 6.5), incubated for 10 min at 37°C and centrifuged at 10,000 *g* for 10 min at 4°C. The absorbance was determined at 370 nm for the tubes with dinitrophenylhydrazine and 280 nm for the tubes with HCl. The results are expressed as concentration of carbonyls (nM)/mg of protein.

### 2.6 Statistical analysis

For factorial design: Two‐way ANOVA and Tukey's multiple comparisons test were conducted utilizing the XLSTAT software (version 2015.4, Addinsoft). A *p*‐value < .05 was considered statistically significant. Principal component analysis (PCA) was used to detect a correlation between treatments with the variable responses. Additionally, a statistical analysis was carried out considering the 10 samples as levels of a single factor to find the differences per day, and a second analysis considering the days as levels of a factor for a single sample.

## 3 RESULTS AND DISCUSSION

### 3.1 Physicochemical characteristics of different treatments of meat

The pH variable presents a significant difference between treatments, days of storage, and type of package (Tables 2 and 3, and Figure S1). Samples stored in permeable packaging were the most susceptible to changes in pH over time compared with vacuum samples. The changes in the control sample (C) and in the avocado extract without encapsulation (AP) are more evident. The less drastic pH changes occurred in the samples with unencapsulated nisin and encapsulated nisin, suggesting that nisin has an important effect on LAB or other microorganisms, inhibiting their growth and, therefore, the decrease in pH by acidification of samples. However, the samples stored under vacuum showed a marked decrease in pH values, which was correlated with LAB growth. It is known that LAB produce lactic acid and other organic acids that induce the pH decrement.

**Table 2:** *p*‐values resulting from statistical analysis of factorial design

	pH	*ɑ*_W_	Oxidized protein	TBARS	Mesophiles	LAB	Coliforms
Principal effects							
A: Day	<0.0001	<0.0001	<0.0001	<0.0001	<0.0001	<0.0001	<0.0001
B: Package	<0.0001	0.6594	<0.0001	0.0142	<0.0001	<0.0001	<0.0001
C: Treatment	<0.0001	0.0099	<0.0001	<0.0001	0.0002	0.0002	0.5667
Interactions							
AB	<0.0001	<0.0001	0.0003	0.4953	<0.0001	0.0023	<0.0001
AC	<0.0001	0.0248	0.0001	0.4525	0.0017	0.0037	0.0007
BC	0.0045	0.0019	0.0174	0.7213	0.6752	0.6842	0.0201
ABC	0.0008	<0.0001	0.4650	0.9738	0.0309	0.0401	0.1772

**Table 3:** pH and *ɑ*_W_ results for the different days and treatments

Treatment	Day				
	1	3	6	8	10
pH					
CP	5.83 ± 0.06^b^	5.30 ± 0.02^CDd^	5.26 ± 0.01^BCDd^	5.46 ± 0.02^BCc^	6.14 ± 0.02^ABa^
AP	5.82 ± 0.07^b^	5.47 ± 0.05^BCb^	5.46 ± 0.02^ABCb^	5.74 ± 0.13^Ab^	6.48 ± 0.14^Aa^
NP	5.83 ± 0.08^ab^	5.73 ± 0.07^Aab^	5.68 ± 0.02^Ab^	5.70 ± 0.04^Aab^	6.00 ± 0.14^Ba^
EMP	5.83 ± 0.06^a^	5.46 ± 0.03^BCb^	5.44 ± 0.06^ABCb^	5.61 ± 0.02^ABb^	6.00 ± 0.01^Ba^
MANP	5.83 ± 0.06	5.57 ± 0.06^AB^	5.48 ± 0.11^AB^	5.72 ± 0.03^A^	5.94 ± 0.27^B^
CV	5.82 ± 0.06^a^	5.20 ± 0.05^Db^	5.12 ± 0.03^Db^	5.19 ± 0.01^DEb^	5.21 ± 0.01^Cb^
AV	5.83 ± 0.07^a^	5.36 ± 0.07^BCDb^	5.21 ± 0.02^BCDbc^	5.15 ± 0.01^DEc^	5.27 ± 0.04^Cbc^
NV	5.83 ± 0.05^a^	5.73 ± 0.08^Aa^	5.35 ± 0.01^BCDb^	5.34 ± 0.01^CDb^	5.42 ± 0.03^Cb^
EMV	5.82 ± 0.06^a^	5.29 ± 0.07^CDb^	5.20 ± 0.01^CDb^	5.20 ± 0.05^DEb^	5.22 ± 0.03^Cb^
MANV	5.83 ± 0.06^a^	5.38 ± 0.09^BCDb^	5.23 ± 0.17^BCDb^	5.07 ± 0.04^Eb^	5.25 ± 0.02^Cb^
*ɑ*_W_					
CP	0.998 ± 0.006^a^	0.995 ± 0.001^CDa^	0.995 ± 0.005^Aa^	0.993 ± 0.00^Ba^	0.985 ± 0.001^DEb^
AP	0.997 ± 0.004^a^	0.994 ± 0.001^Da^	0.994 ± 0.001^Aa^	0.994 ± 0.001^Aa^	0.986 ± 0.003^Db^
NP	0.998 ± 0.006^a^	0.995 ± 0.001^CDa^	0.989 ± 0.001^BCab^	0.990 ± 0.001^Dab^	0.985 ± 0.003^Eb^
EMP	0.998 ± 0.005^a^	0.994 ± 0.001^Dab^	0.991 ± 0.002^Bab^	0.987 ± 0.001^GHbc^	0.982 ± 0.003^Fc^
MANP	0.997 ± 0.007^a^	0.995 ± 0.002^CDa^	0.988 ± 0.001^CDb^	0.987 ± 0.001^Gb^	0.986 ± 0.001^DEb^
CV	0.997 ± 0.008^ab^	0.997 ± 0.001^Ba^	0.984 ± 0.00^Fc^	0.989 ± 0.002^Ebc^	0.996 ± 0.001^Aa^
AV	0.998 ± 0.006^ab^	0.996 ± 0.001^BCa^	0.985 ± 0.001^EFc^	0.986 ± 0.002^Hc^	0.989 ± 0.001^Cbc^
NV	0.998 ± 0.006^a^	0.995 ± 0.002^CDab^	0.986 ± 0.002^EFc^	0.988 ± 0.002^EFbc^	0.993 ± 0.001^Bab^
EMV	0.997 ± 0.008^a^	0.994 ± 0.001^Da^	0.986 ± 0.001^DEb^	0.992 ± 0.001^Cab^	0.992 ± 0.001^Bab^
MANV	0.998 ± 0.005^ab^	0.998 ± 0.002^Aa^	0.991 ± 0.002^Bbc^	0.988 ± 0.002^FGcd^	0.983 ± 0.001^Fd^

Abbreviations: AP, Avocado peel extract‐permeable; AV, Avocado peel extract‐vacuum; CP, control‐permeable; CV, control‐vacuum; EMP, empty microcapsule‐permeable; EMV, empty microcapsule‐vacuum; MANP, microcapsule with avocado peel extract and nisin‐permeable; MANV, microcapsule with avocado peel extract and nisin‐vacuum; NP, nisin‐permeable; NV, nisin‐vacuum.

A–H: significant difference between treatments in the same day (*p* < .05), a–e: significant difference between days in the same treatment (*p* < .05).

In contrast, *ɑ*_W_ values decreased as a function of time (Tables 2 and 3), and the treatments with microcapsules, as well as the permeable package samples, were the most affected. The decrease in *ɑ*_W_ values could be related to the microcapsules’ capacity for holding water with some polysaccharides, such as pectin, for its functional groups (Vaclavik & Christian, 2014). These characteristics of polysaccharides, plus the presence of the extract and nisin, might be associated with the lower microbial load for treated samples.

Both pH and *ɑ*_W_, as well as other parameters such as temperature, are factors that can destabilize the microcapsules made by the coacervation method because these factors are controlled during their elaboration and allow for interaction between the polymers that make them, thus affecting the release of the encapsulated bioactive compound (Qv, Zeng, & Jiang, 2011).

#### 3.1.1 Oxidation stability

The three study factors (type of package, treatment, and time) have an important effect on oxidation (Table 2). The samples with treatments that contain avocado peel extract (alone and encapsulated) have lower TBARS. The oxidation of vacuum‐packaged samples was lower than those in the permeable packaging due to the decrease in oxygen content (Table 4). Similar behavior was presented on protein oxidation (Tables 2 and 4), where the oxidation was inferior in the samples that have microcapsules with avocado peel extract and nisin, as well as in the extract without encapsulation. The presence of polyphenols in the avocado peel extract (around of 19.7 ± 1 mg equivalents of gallic acid/g of extract), such as epicatechin, isorhamnetin, and kaempferide epicatechin gallate, among others (Calderón‐Oliver et al., 2016), prevent or decrease oxidation in minced meat. It is well known that polyphenols, according to their structure and concentration, have antioxidant properties via different mechanisms (Leopoldini, Russo, & Toscano, 2011).

**Table 4:** TBARS and oxidized protein results for the different days and treatments

Treatment	Day				
	1	3	6	8	10
TBARS (mg MDA/kg)					
CP	0.125 ± 0.035^c^	0.199 ± 0.001^b^	0.238 ± 0.003^ab^	0.241 ± 0.001^ABab^	0.277 ± 0.004^a^
AP	0.123 ± 0.030^b^	0.142 ± 0.011^b^	0.157 ± 0.033^ab^	0.162 ± 0.003^Cab^	0.247 ± 0.024^a^
NP	0.124 ± 0.035	0.207 ± 0.062	0.191 ± 0.083	0.189 ± 0.016^ABC^	0.260 ± 0.013
EMP	0.125 ± 0.033^b^	0.192 ± 0.054^ab^	0.181 ± 0.027^ab^	0.255 ± 0.006^Aa^	0.241 ± 0.044^ab^
MANP	0.124 ± 0.038^b^	0.137 ± 0.018^ab^	0.160 ± 0.001^ab^	0.159 ± 0.001^Cab^	0.203 ± 0.057^a^
CV	0.126 ± 0.030^b^	0.191 ± 0.008^ab^	0.209 ± 0.013^ab^	0.227 ± 0.001^ABCab^	0.261 ± 0.029^a^
AV	0.123 ± 0.032	0.129 ± 0.030	0.142 ± 0.012	0.165 ± 0.021^C^	0.241 ± 0.005
NV	0.125 ± 0.038	0.163 ± 0.053	0.154 ± 0.023	0.171 ± 0.041^BC^	0.230 ± 0.005
EMV	0.123 ± 0.035	0.155 ± 0.064	0.154 ± 0.065	0.168 ± 0.028^C^	0.247 ± 0.005
MANV	0.126 ± 0.030^b^	0.111 ± 0.013^b^	0.116 ± 0.021^b^	0.169 ± 0.002^Cab^	0.227 ± 0.035^a^
Protein oxidation (carbonyls nM/mg protein)					
CP	0.375 ± 0.035^e^	0.840 ± 0.057^d^	1.300 ± 0.141^c^	1.750 ± 0.071^Ab^	2.350 ± 0.071^ABa^
AP	0.345 ± 0.038^c^	0.517 ± 0.024^bc^	0.940 ± 0.085^abc^	1.250 ± 0.354^ABab^	1.650 ± 0.212^BCa^
NP	0.365 ± 0.030^c^	0.735 ± 0.191^c^	1.400 ± 0.141^b^	1.775 ± 0.106^Ab^	2.400 ± 0.141^Aa^
EMP	0.348 ± 0.025^e^	0.730 ± 0.042^d^	1.300 ± 0.141^c^	1.750 ± 0.071^Ab^	2.350 ± 0.071^ABa^
MANP	0.370 ± 0.020^c^	0.525 ± 0.035^c^	0.800 ± 0.025^b^	1.100 ± 0.141^ABa^	1.200 ± 0.003^Ca^
CV	0.365 ± 0.035^c^	0.575 ± 0.106^bc^	0.850 ± 0.071^bc^	1.350 ± 0.212^ABab^	1.750 ± 0.354^ABCa^
AV	0.365 ± 0.030^c^	0.425 ± 0.035^c^	0.750 ± 0.071^b^	1.000 ± 0.005^Bb^	1.400 ± 0.141^Ca^
NV	0.372 ± 0.025^c^	0.625 ± 0.035^bc^	1.050 ± 0.354^abc^	1.400 ± 0.283^ABab^	1.600 ± 0.005^Ca^
EMV	0.370 ± 0.030^c^	0.685 ± 0.262^bc^	1.250 ± 0.354^abc^	1.350 ± 0.212^ABab^	1.750 ± 0.071^ABCa^
MANV	0.368 ± 0.030^b^	0.425 ± 0.035^b^	0.825 ± 0.035^ab^	0.900 ± 0.009^Bab^	1.250 ± 0.354^Ca^

Abbreviations: AP, Avocado peel extract‐permeable; AV, Avocado peel extract‐vacuum; CP, control‐permeable; CV, control‐vacuum; EMP, empty microcapsule‐permeable; EMV, empty microcapsule‐vacuum; MANP, microcapsule with avocado peel extract and nisin‐permeable; MANV, microcapsule with avocado peel extract and nisin‐vacuum; NP, nisin‐permeable; NV, nisin‐vacuum.

A–C: significant difference between treatments in the same day (*p* < .05), a–c: significant difference between days in the same treatment (*p* < .05).

Interestingly, on day 10, the combination of empty packaging with the preservative decreased the oxidation of the proteins by ~46% compared with that of the control stored in permeable packaging and by ~28.5% compared with the vacuum control, suggesting the effectiveness of the microcapsules in preventing oxidation and that this effect is much stronger with vacuum storage.

Similar results were observed in meat patties, in which avocado peel extract reduced around 60% of the protein oxidation and 20% of the lipid oxidation of the system (Rodríguez‐Carpena, Morcuende, & Estévez, 2011). However, there are other studies in which agro‐industrial residues that contain polyphenols decrease the oxidation in meat and meat products (Monteiro et al., 2014; Zhang, 2015). Surprisingly, when encapsulated by other techniques (e.g., spray drying), the activity of these extracts decreases or changes. This is the case for the extract of Jabuticaba peel, which does not induce a significant decrease in oxidation levels when applied on mortadella (Baldin et al., 2018).

The decrease in the oxidation of lipids and proteins can translate into an extended shelf life for the product, as well as a reduction in sensory changes such as rancidity.

### 3.2 Microbiological stability of meat with microcapsules

The storage time, type of packaging, and presence of microcapsules show a significant (*p* < .05) (Table 2) effect on microbial growth. In general, the microbial populations increased over time (Table 5 and Figure S2). The meat samples with microcapsules that contain avocado seed extract and nisin presented a lower population of mesophilic bacteria, compared with other samples. Additionally, vacuum‐packaged samples presented lower mesophilic counts than those with a permeable package. The importance in the counting of these bacteria is that the presence of them can contribute to the deterioration of the meat and sensory changes, since these bacteria can have proteolytic and lipolytic enzymes, as is the case for *Carnobacterium*, *Serratia,* and *Pseudomonas* (Ercolini, Russo, Nasi, Ferranti, & Villani, 2009); those changes can be prevented by the addition of microcapsules in combination with vacuum packaging.

**Table 5:** Microbiological results for the different days and treatments

Treatment	Day				
	1	3	6	8	10
Mesophiles (Log CFU/g)					
CP	4.23 ± 0.21^d^	5.48 ± 0.09^Acd^	6.47 ± 0.28^bc^	7.17 ± 0.10^b^	11.02 ± 0.65^ABCa^
AP	4.73 ± 0.20^c^	5.61 ± 0.01^Ac^	5.12 ± 0.71^bc^	7.87 ± 0.43^b^	12.18 ± 1.03^Aa^
NP	4.33 ± 0.31^c^	5.59 ± 0.38^Abc^	5.95 ± 0.54^bc^	8.21 ± 0.02^b^	11.55 ± 1.5^ABa^
EMP	4.25 ± 0.20^e^	5.55 ± 0.07^Ad^	6.51 ± 0.07^c^	8.69 ± 0.27^b^	10.83 ± 0.08^ABCa^
MANP	4.72 ± 0.18^d^	4.73 ± 0.28^Bd^	6.16 ± 0.68^c^	7.27 ± 1.08^b^	10.54 ± 0.18^ABCa^
CV	4.52 ± 0.20^d^	5.34 ± 0.15^Ac^	5.87 ± 0.01^c^	7.35 ± 0.35^b^	9.58 ± 0.02^BCDa^
AV	4.23 ± 0.10^c^	5.50 ± 0.21^Abc^	5.99 ± 0.01^b^	6.81 ± 0.78^b^	9.66 ± 0.29^BCDa^
NV	4.52 ± 0.20^d^	5.45 ± 0.46^Acd^	6.32 ± 0.72^bc^	7.07 ± 0.09^b^	8.82 ± 0.10^CDa^
EMV	4.60 ± 0.12^d^	5.51 ± 0.03^Ac^	6.21 ± 0.43^c^	7.69 ± 0.27^b^	9.63 ± 0.05^BCDa^
MANV	4.55 ± 0.21^d^	5.05 ± 0.26^Acd^	6.07 ± 0.76^bc^	7.12 ± 0.01^ab^	7.75 ± 0.10^Da^
Coliforms (Log CFU/g)					
CP	2.95 ± 0.14^c^	4.01 ± 0.16^ABbc^	4.62 ± 0.08^b^	4.89 ± 0.58^b^	10.14 ± 0.01^a^
AP	2.97 ± 0.16^d^	4.07 ± 0.04^ABcd^	4.34 ± 0.44^bc^	5.38 ± 0.54^b^	9.75 ± 0.21^a^
NP	2.96 ± 0.21^c^	3.77 ± 0.38^ABbc^	4.35 ± 0.49^b^	4.79 ± 0.01^b^	9.71 ± 0.01^a^
EMP	2.95 ± 0.38^d^	4.04 ± 0.05^ABcd^	4.61 ± 0.25^c^	6.18 ± 0.53^b^	9.54 ± 0.11^a^
MANP	2.85 ± 0.20^c^	3.65 ± 0.04^Bc^	4.75 ± 0.92^b^	5.76 ± 0.37^b^	9.54 ± 0.05^a^
CV	2.95 ± 0.22^d^	4.27 ± 0.05^ABc^	4.80 ± 0.01^bc^	5.29 ± 0.29^b^	6.60 ± 0.03^a^
AV	2.90 ± 0.20^c^	4.50 ± 0.10^Ab^	4.82 ± 0.10^b^	5.25 ± 0.49^b^	6.54 ± 0.01^a^
NV	2.92 ± 0.18^d^	4.05 ± 0.21^ABcd^	5.02 ± 0.64^bc^	5.74 ± 0.61^b^	8.00 ± 0.02^a^
EMV	2.95 ± 0.20^d^	4.21 ± 0.21^ABc^	5.23 ± 0.01^b^	5.76 ± 0.46^ab^	6.48 ± 0.02^a^
MANV	2.96 ± 0.20^c^	4.08 ± 0.03^ABbc^	5.07 ± 0.56^ab^	5.79 ± 0.41^a^	6.30 ± 0.01^a^
LAB (Log CFU/g)					
CP	4.36 ± 0.18^d^	4.49 ± 0.02^BCd^	4.85 ± 0.10^ABc^	5.36 ± 0.06^ABb^	8.40 ± 0.20^ABa^
AP	4.30 ± 0.16^c^	4.85 ± 0.08^Bbc^	4.70 ± 0.07^ABbc^	4.94 ± 0.09^ABb^	8.76 ± 0.23^ABa^
NP	4.26 ± 0.10^b^	4.26 ± 0.02^Cb^	4.25 ± 0.07^Bb^	4.45 ± 0.07^Bb^	8.13 ± 0.75^Ba^
EMP	4.36 ± 0.18^d^	4.80 ± 0.10^Bc^	5.07 ± 0.10^ABbc^	5.33 ± 0.04^ABb^	8.35 ± 0.07^ABa^
MANP	4.30 ± 0.21^b^	4.27 ± 0.07^Cb^	4.88 ± 0.53^ABb^	5.07 ± 0.38^ABb^	7.92 ± 0.17^Ba^
CV	4.35 ± 0.18^d^	4.84 ± 0.10^Bcd^	5.29 ± 0.01^ABbc^	5.50 ± 0.01^ABb^	9.19 ± 0.27^ABa^
AV	4.40 ± 0.20^c^	5.75 ± 0.10^Ab^	5.50 ± 0.01^ABbc^	5.39 ± 0.01^ABbc^	8.60 ± 0.71^ABa^
NV	4.30 ± 0.15^b^	4.58 ± 0.09^BCb^	5.17 ± 0.39^ABb^	5.21 ± 0.29^ABb^	9.09 ± 0.04^ABa^
EMV	4.35 ± 0.18^c^	4.71 ± 0.11^Bc^	5.65 ± 0.07^Ab^	5.60 ± 0.01^ABb^	9.65 ± 0.21^Aa^
MANV	4.40 ± 0.18^b^	4.77 ± 0.22^Bb^	5.05 ± 0.78^ABb^	6.07 ± 0.08^Ab^	8.59 ± 0.01^ABa^

Abbreviations: AP, Avocado peel extract‐permeable; AV, Avocado peel extract‐vacuum; CP, control‐permeable; CV, control‐vacuum; EMP, empty microcapsule‐permeable; EMV, empty microcapsule‐vacuum; MANP, microcapsule with avocado peel extract and nisin‐permeable; MANV, microcapsule with avocado peel extract and nisin‐vacuum; NP, nisin‐permeable; NV, nisin‐vacuum.

A–D: significant difference between treatments in the same day (*p* < .05), a–d: significant difference between days in the same treatment (*p* < .05).

The two treatments with nisin presented a lower LAB population. This was expected, as one of the main targets of this bacteriocin are closely related LAB and other Gram‐positive bacteria (Delves‐Broughton & Weber, 2011). However, the count of lactic acid bacteria was greater in vacuum‐packaged samples because these bacteria are facultative anaerobes (Batt, 2014). Some bacteria that grow in vacuum‐packaged meat, such as *Lactococcus lactis*, *Pediococcus acidilactici,* and different species of *Lactobacillus*, can inhibit the growth of other bacteria and contribute to the increased shelf life of a product (Oliveira, Oliveira, & Glória, 2008) because they can produce bacteriocins with different mechanisms of action (Cleveland, Montville, Nes, & Chikindas, 2001). There is a slight tendency for treatment with encapsulated nisin to decrease the growth of LAB over time, and this could be the result of a gradual release of nisin into the medium.

In contrast, the presence of microcapsules did not affect the coliform bacterial population. However, the permeable storage samples presented greater coliform counts.

Samples containing empty microcapsules showed slightly higher microbial populations that may be associated with the presence of microbial enzymes that allow for the metabolization of pectin and other complex polysaccharides, such as species of *Lactobacillus*, *Bacillus*, among others of the genus enterobacteria (Abbott & Boraston, 2008; Jayani, Saxena, & Gupta, 2005). This slight increase in the microbial population by empty microcapsules could be reduced if it is mixed with some other barrier method, as explained by Baldin et al., (2018) who incorporated microcapsules with antioxidant extract in mortadella sausages, where thermal treatment helped to reduce the microbial count.

The antimicrobial activity presented by microcapsules, besides nisin, is related to their polyphenol content on AE. The general mechanism by which polyphenols exhibit antimicrobial activity is broad, from the inhibition of the synthesis of the cell wall, change in the permeability of the membrane and cell wall, even inhibiting the formation of biofilms and mobility, or polyphenols can inhibit enzymes or regulate the expression of some genes (Papuc, Goran, Predescu, Nicorescu, & Stefan, 2017). Microcapsules made by a complex or simple coacervation method present a good release rate of antimicrobial compounds compared with other encapsulation methods, and this depends on the final morphology, surface area, concentration of the antimicrobial agent and dispersion system (Castro‐Rosas et al., 2017).

### 3.3 Principal component analysis (PCA) of different meat treatments and overall overview

**Figure 1:**
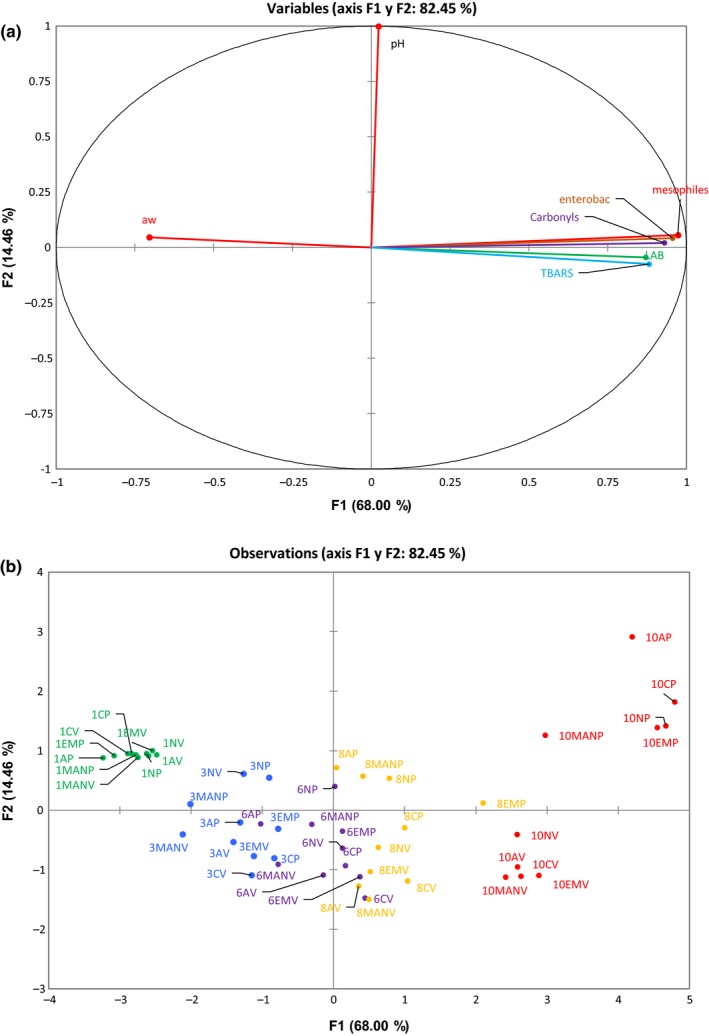
Principal component analysis. (a) Response variables distribution. (b) Treatment distribution. The number at the beginning indicates the storage day, the next letters indicates the treatment and package: AP, Avocado peel extract‐permeable; AV, Avocado peel extract‐vacuum; CP, control‐permeable; CV, control‐vacuum; EMP, empty microcapsule‐permeable; EMV, empty microcapsule‐vacuum; MANP, microcapsule with avocado peel extract and nisin‐permeable; MANV, microcapsule with avocado peel extract and nisin‐vacuum; NP, nisin‐permeable; NV, nisin‐vacuum

Principal component analysis was performed to study which variable responses were correlated or best describe each treatment. In Figure 1a,b, factor 1 (*x*‐axis) explains 68% of the total variability in the data. This means that the variable responses, such as bacteria count, oxidation of macromolecules, and *ɑ*_W_, were correlated in 68% of the samples. The microbiological and oxidative variable responses describe the last days of storage (day 8 and 10), while the first and third day were positively correlated with the *ɑ*_W_ response. The main differences between treatments were marked by differences in bacterial growth and oxidation, which are important for estimating product quality. Factor 2, which explains 14.46% of the variance, separates the samples as a function of the pH, and there is a tendency for the treatments with permeable packages to be correlated with the pH values. The correlation is more evident at the end of storage time.

The samples with microcapsules containing nisin and avocado extract present a slight tendency to behave similar to the samples from the analysis on the previous day, by decreasing the oxidation of lipids and proteins and microbial growth.

## 4 CONCLUSION

The addition of microcapsules with an antioxidant and an antimicrobial effect on minced meat, such as avocado peel extract and nisin, decreases the oxidation of lipids and proteins and decreases the growth of bacteria such as mesophiles and BAL. These findings indicate that microcapsules increase the effect of antimicrobial and antioxidant properties in the minced meat, and its effect is better when combined with other technology such as vacuum packaging. These microcapsules could be used as natural preservatives in the food industry to reduce the concentration of some preservatives or eliminate the use of synthetic preservatives.

## CONFLICT OF INTEREST

The authors declare that they have no conflict of interest.

## ETHICAL APPROVAL

The study did not involve any human or animal testing.
