# Supplementary figures and images for: Effect of the addition of microcapsules with avocado peel extract and nisin on the quality of ground beef
Source: Food Sci Nutr. 2020 Feb 22;8(3):1325–34. doi: 10.1002/fsn3.1359 (PMC7063373; doi:10.1002/fsn3.1359)

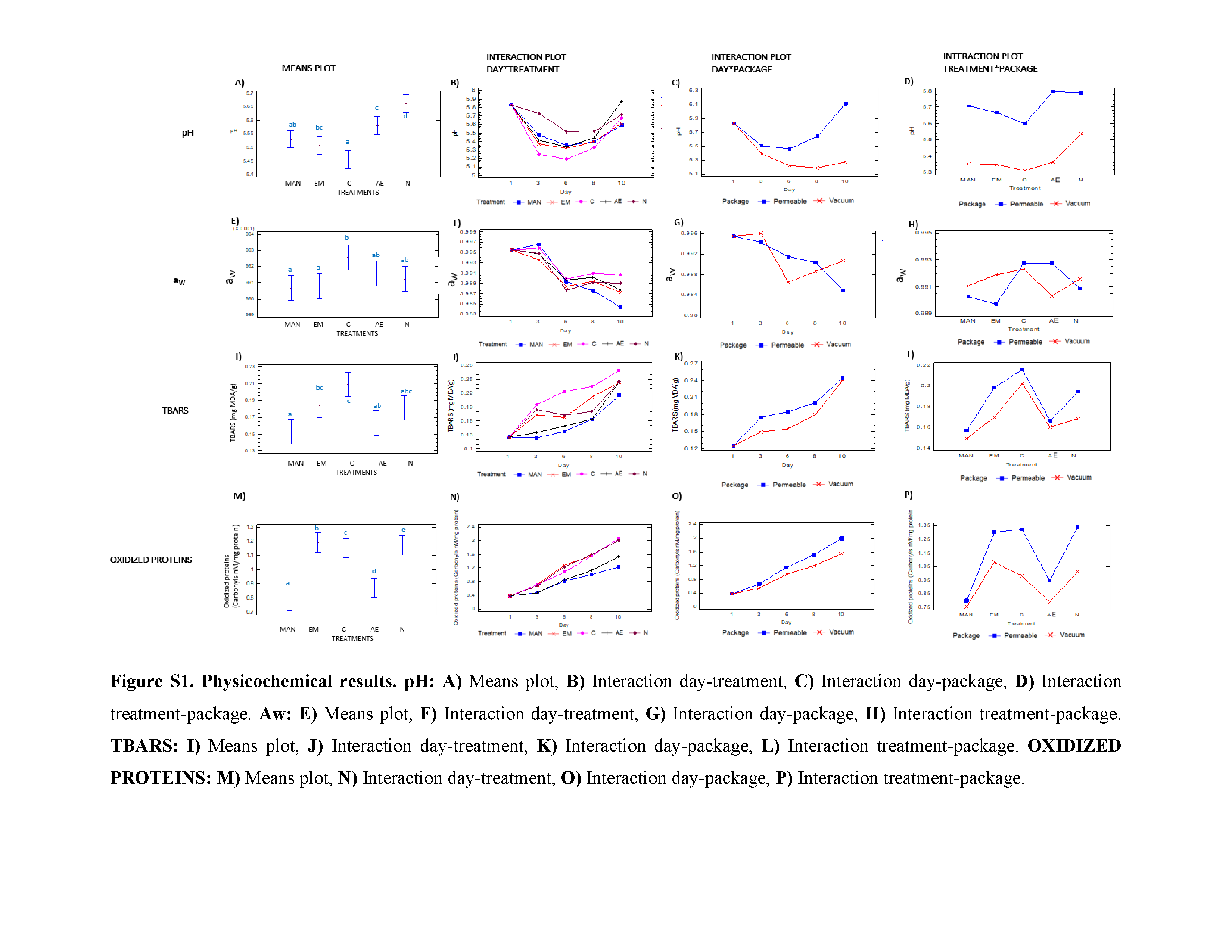

Supplement: Supplementary file 1 [file FSN3-8-1325-s001.tif]

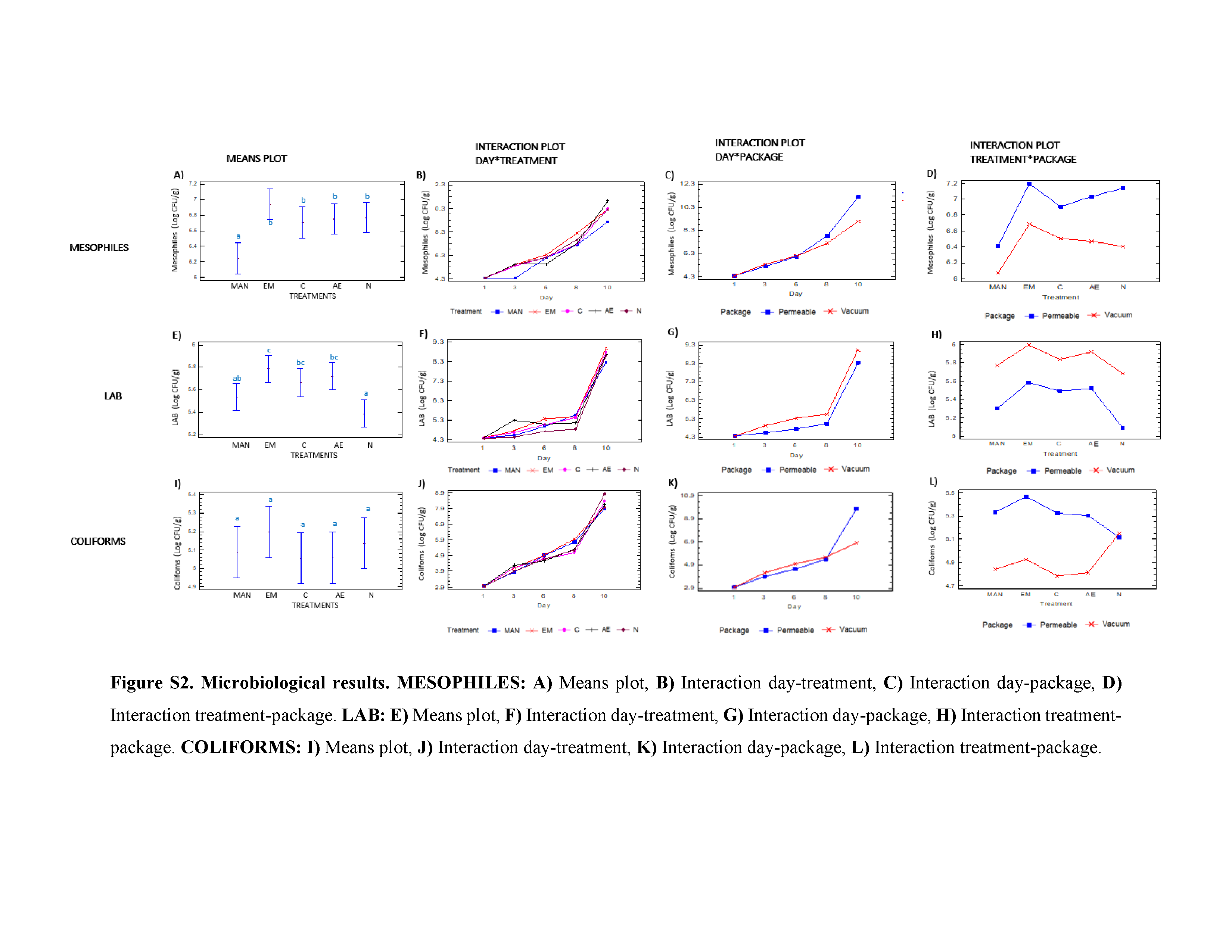

Supplement: Supplementary file 2 [file FSN3-8-1325-s002.tif]
